# Crystal structure and Hirshfeld surface analysis of (benzo[4,5]imidazo[2,1-*b*]thia­zol-2-yl)(4-chlorophen­yl)methanone

**DOI:** 10.1107/S2056989026010091

**Published:** 2026-10-02

**Authors:** R. Vishal, Vignesh Balaraman, Rajesh B. R. D. Yamajala, Sampath Natarajan

**Affiliations:** a Chemical Biology Lab, School of Chemical and Biotechnology, SASTRA Deemed University, Tirumalaisumudiram, Thanjavur, Tamil Nadu-613401, India; b Organic Synthesis & Catalysis Lab, Department of Chemistry, School of Chemical, and Biotechnology, SASTRA Deemed University, Thanjavur – 613 401, Tamil Nadu, India; University of Hyogo, Japan

**Keywords:** crystal structure, Hirshfeld surface, monoclinic, hydrogen bond, benzimidazole

## Abstract

Thia­zolo[3,2-*a*]benzimidazole derivatives are biologically important heterocycles with diverse pharmacological activities such as anti­cancer and anti-inflammatory. The compound, C_16_H_9_ClN_2_OS, crystallizes in the monoclinic space group *P*2_1_/*c* with two conformationally similar mol­ecules (*Z*′ = 2) in the asymmetric unit. The fused thia­zolo[3,2-*a*]benzimidazole framework is essentially planar, while the 4-chloro­benzoyl ring is inclined by ∼53° to the thia­zole ring in both mol­ecules. The crystal packing is consolidated by C—H⋯N hydrogen bonds, C—H⋯π contacts, and π–π stacking inter­actions, forming a three-dimensional supra­molecular framework. Hirshfeld surface analysis indicates that H⋯H, H⋯C/C⋯H, H⋯Cl/Cl⋯H, H⋯N/N⋯H, and H⋯O/O⋯H contacts dominate the inter­molecular inter­actions.

## Abstract 2

The title compound crystallizes in the monoclinic space group *P*2_1_/*c* with two conformationally similar mol­ecules (*Z*′ = 2) in the asymmetric unit. The fused thia­zolo[3,2-*a*]benzimidazole framework is essentially planar, while the 4-chloro­benzoyl ring is inclined by ∼53° to the thia­zole ring in both mol­ecules. The crystal packing is consolidated by C—H⋯N hydrogen bonds, C—H⋯π contacts, and π–π stacking inter­actions, forming a three-dimensional supra­molecular framework.

## 1. Chemical context

Benzimidazole is an important nitro­gen-containing heterocyclic scaffold with a broad spectrum of biological activities, including anti­cancer, anti­microbial, and anti-inflammatory properties (Anichina *et al.*, 2017 ▸; Ramos Rodríguez *et al.*, 2019 ▸). Fusion with a thia­zole ring affords thia­zolo[3,2-*a*]benzimidazole derivatives, which often exhibit enhanced pharmacological profiles and have shown promise against cancer, HIV, and microbial infections (Al-Rashood & Abdel-Aziz, 2010 ▸; Khaldi *et al.*, 2020 ▸). Given that substitution patterns on the fused ring system significantly influence bioactivity, structural characterization by single-crystal X-ray diffraction is essential for establishing the mol­ecular geometry and crystal packing, which are crucial for understanding structure–activity relationships (Ramos Rodríguez *et al.*, 2020 ▸). As part of our ongoing research on bioactive heterocycles (Al-Ansary *et al.*, 2017 ▸), we report the crystal structure of the title compound, C_16_H_9_ClN_2_OS, (**3**).
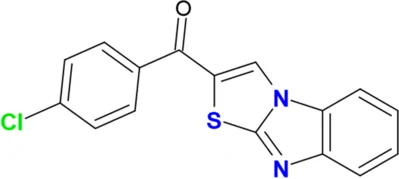


## 2. Structural commentary

The title compound crystallizes in the monoclinic space group *P*2_1_/*c* with two crystallographically independent mol­ecules, *A* and *B*, in the asymmetric unit (Fig. 1 ▸). Both mol­ecules share the same fused thia­zolobenzimidazole core, comprising a thia­zole ring (*B*), an imidazole ring (*C*), and a benzene ring (*D*), which adopts an essentially planar conformation in both independent mol­ecules as observed in similar reported structures (Fun *et al.*, 2012*a* ▸,*b* ▸; Wang *et al.*, 2011 ▸). However, a comparative analysis reveals subtle differences in the degree of planarity between the two mol­ecules: the inter­planar angles between rings *B*/*C*, *C*/*D*, and *B*/*D* are 1.2 (2), 1.7 (1), and 2.7 (2)° for mol­ecule *A*, compared to 1.0 (1), 0.8 (2), and 1.3 (2)° for mol­ecule *B*, indicating that mol­ecule *B* exhibits marginally greater coplanarity of the fused ring system, as further supported by the corresponding torsion angles (see the torsion angle table in the supporting information). In contrast, the 4-chloro­benzoyl substituents attached at the C9 position of the thia­zole ring show a significant and remarkably similar deviation from the fused ring plane in both mol­ecules, with dihedral angles between the 4-chloro­phenyl ring (*A*) and the thia­zole ring (*B*) of 53.0 (1)° for mol­ecule *A* and 53.1 (3)° for mol­ecule *B*, suggesting that this conformational preference is primarily governed by intrinsic electronic factors and steric avoidance rather than crystal packing influences. All bond lengths and bond angles (Table 1 ▸) in both mol­ecules are within the expected ranges for analogous heterocyclic systems and show no statistically significant differences between the two independent mol­ecules, confirming that mol­ecules *A* and *B* are chemically equivalent but exhibit minor conformational variations, particularly in the planarity of the fused thia­zolobenzimidazole core.

## 3. Supra­molecular features and Hirshfeld surface analysis

Fig. 2 ▸ illustrates the packing of structure **3** along the crystallographic *a*-axis direction, wherein the overall cohesion of the unit cell is sustained by two inter­molecular hydrogen bonds (Table 2 ▸; Fig. 3 ▸), C8*A*—H8*A*⋯N1*A* and C8*B*—H8*B*⋯N1*B*, that propagate into supra­molecular chains parallel to the *c* axis. The crystal packing is characterized by weak C—H⋯π and aromatic π–π contacts (Fig. 4 ▸). The C13*B*—H13*B*⋯*Cg*1 and C16*A*—H16*A*⋯*Cg*7 contacts provide additional inter­molecular links between neighbouring mol­ecules. Several pairs of aromatic rings exhibit centroid-to-centroid separations in the range 3.731 (2)–4.011 (2) Å, namely *Cg*1⋯*Cg*3 = 3.731 (2) Å, *Cg*2⋯*Cg*3 = 4.011 (2) Å, *Cg*3⋯*Cg*4 = 3.748 (2) Å, *Cg*5⋯*Cg*7 = 3.893 (2) Å, *Cg*6⋯*Cg*7 = 3.924 (2) Å and *Cg*7⋯*Cg*8 = 3.787 (2) Å where *Cg*1, *Cg*2, *Cg*3 and *Cg*4 denote the centroids of the S1*A*/C7*A*/N2*A*/C8*A*/C9*A*, N1*A*/C1*A*/C6*A*/N2*A*/C7*A*, C11*A*–C16*A* and S1*A*/C7*A*/N1*A*/C1*A*/C6*A*/N2*A*/C8*A/*C9*A* ring systems, respectively, while *Cg*5, *Cg*6, *Cg*7 and *Cg*8 denote the corresponding centroids of the S1*B/*C7*B*/N2*B*/C8*B*/C9*B*, N1*B*/C1*B*/C6*B*/N2*B*/C7*B*, C1*B*–C6*B* and S1*B*/C7*B/*N1*B*/C1*B*/C6*B*/N2*B*/C8*B*/C9*B* ring systems. The *Cg*1⋯*Cg*3^i^, *Cg*3⋯*Cg*4^i^ and *Cg*7⋯*Cg*8^ii^ contacts are consistent with weak aromatic π–π associations (Fig. 4 ▸), while the *Cg*5⋯*Cg*7^ii^ and *Cg*6⋯*Cg*7^ii^ contacts, with slightly larger centroid-to-centroid separations, are indicative of weaker slipped or offset aromatic contacts [symmetry codes: (i) *x*, 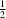 − *y*, 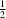 + *z*; (ii) *x*, 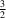 − *y*, −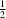 + *z*]. The *Cg*2⋯*Cg*3^i^ separation of 4.011 (2) Å is comparatively long and is therefore regarded as a weak or borderline aromatic contact rather than a conventional π–π stacking inter­action. Thus, the observed contacts are not inter­preted as idealized face-to-face π–π stacks, but rather as a range of weak, slipped or offset aromatic associations arising from the relative disposition of the ring systems in the crystal. Together with the C—H⋯π contacts, these inter­actions contribute to the overall supra­molecular organization of the crystal. Overall, these weak aromatic contacts, together with the other inter­molecular inter­actions present in the crystal, contribute to the cohesion and packing of the mol­ecular structure.

The Hirshfeld surfaces (Fig. 5 ▸) and corresponding two-dimensional fingerprint plots (Fig. 4 ▸) of mol­ecules *A* and *B* provide a comparative view of their inter­molecular contacts and crystal-packing characteristics (Spackman *et al.*, 2021 ▸). In both mol­ecules, the *d*_norm_ mapped surfaces show localized red regions associated with close inter­molecular contacts, particularly around the heteroatom-containing sites. The Hirshfeld fingerprint analysis (Fig. 6 ▸) reveals closely comparable inter­molecular contact distributions for the two independent mol­ecules. For mol­ecule *A*, the major contributions arise from C⋯H/H⋯C (21.4%), H⋯H (19.3%), Cl⋯H/H⋯Cl (13.0%), N⋯H/H⋯N (10.9%) and O⋯H/H⋯O (10.2%) contacts, while the corresponding contributions for mol­ecule *B* are 22.8, 19.4, 10.7, 10.2 and 11.2%, respectively. The fingerprint plots are consistent with these values and show that C⋯H/H⋯C and H⋯H contacts constitute the principal contributions in both mol­ecules. The short C8*A*—H8*A*⋯N1*A* [3.206 (3) Å] and C8*B*—H8*B*⋯N1*B* [3.212 (3) Å] contacts are consistent with the comparable N⋯H/H⋯N contributions. The O⋯H/H⋯O contribution is slightly higher for mol­ecule *B*, consistent with the closer C—H⋯O contact observed for this mol­ecule, whereas mol­ecule *A* exhibits relatively larger Cl⋯H/H⋯Cl and S-associated contributions. Overall, the highly similar fingerprint profiles indicate comparable inter­molecular contact environments for mol­ecules *A* and *B*. The crystal packing is therefore consolidated by a cooperative combination of dispersive H⋯H, C⋯H/H⋯C and heteroatom-associated contacts, rather than by any single dominant inter­action. The small differences in individual contact contributions reflect variations in the local packing environments of the two crystallographically independent mol­ecules.

## 4. Database survey

A systematic analysis of the Cambridge Structural Database (CSD, Version 5.47; Groom *et al.*, 2016 ▸), performed using the CCDC CONQUEST inter­face, was carried out to identify crystallographically characterized compounds structurally related to the title compound **3**. The search identified two closely related derivatives, namely benzo[4,5]imidazo[2,1-*b*]thia­zol-2-yl(*p*-tol­yl)methanone (CSD refcode ABESUY) and benzo[4,5]imidazo[2,1-*b*]thia­zol-2-yl(4-fluoro­phen­yl)methan­one (ABESOS), reported by Ambethkar *et al.* (2017 ▸). These compounds contain *para*-methyl and *para*-fluoro substituents, respectively, on the phenyl ring of the benzoyl group. Their presence in the CSD provides useful structural precedents for assessing the influence of substitution on the conformation of the benzoyl-containing thia­zolobenzimidazole framework.

The CSD search was subsequently extended to related compounds containing the thia­zolo[3,2-*a*]benzimidazole core. Among the relevant structures, BACCUH (Chen *et al.*, 2020 ▸) and AMUCIW (Yallur *et al.*, 2016 ▸) are particularly useful for comparison. In these compounds, the benzoyl functionality is replaced by an aldehyde group; nevertheless, the principal geometrical characteristics and conformational features of the fused heteroaromatic framework are broadly comparable with those observed for compound **3**. Both structures also display aromatic *π–π* stacking inter­actions, indicating that such contacts may contribute significantly to the supra­molecular organization of thia­zolo[3,2-*a*]benzimidazole derivatives in the solid state. Further structural comparisons can be made with the benzoyl-containing CSD entries CANHEI (Zhang *et al.*, 2021 ▸) and CANHEI01 (Chen *et al.*, 2022 ▸). These structures possess an unsubstituted benzoyl group and exhibit geometrical parameters broadly consistent with those determined for compound **3**. The comparison suggests that modification of the peripheral substituents does not substanti­ally alter the geometry of the fused thia­zolo[3,2-*a*]benzimidazole skeleton, although differences in the orientation of the pendant phenyl ring can occur.

The methyl-ketone derivative FAQPAQ (Abdel-Aziz *et al.*, 2010 ▸) represents another structurally relevant example. In this compound, the thia­zolo[3,2-*a*]benzimidazole unit adopts an essentially planar conformation and is associated with a pyrazolo­[1,5-*a*]pyrimidine system. Although its overall mol­ecular constitution differs from that of compound **3**, its nearly planar heteroaromatic framework supports the conformational preference observed for the corresponding core in the present structure. The reported biological activity of FAQPAQ, including its *in vitro* anti­cancer activity against colon cancer, further illustrates the continued inter­est in this heterocyclic scaffold. Other related structures, such as EXIVOX (Wang *et al.*, 2011 ▸) and QECZAB (Fun *et al.*, 2012*b* ▸), provide additional insight into the inter­molecular organization of this class of compounds. In EXIVOX, no significant classical inter­molecular hydrogen-bonding inter­actions were reported, whereas the crystal packing of QECZAB is substanti­ally influenced by *π–π* stacking inter­actions. These observations are consistent with the possibility that aromatic stacking and other weak inter­molecular contacts can play an important role in consolidating the crystal packing of thia­zolo[3,2-*a*]benzimidazole derivatives.

Overall, the CSD comparison indicates that the fused thia­zolo[3,2-*a*]benzimidazole core generally favours an approximately planar geometry, consistent with its extended conjugated aromatic system. In compound **3**, however, attachment of the benzoyl substituent introduces an additional torsional degree of freedom, allowing the pendant phenyl ring to rotate with respect to the heteroaromatic core. The magnitude of this torsional deviation is likely governed by a combination of steric and electronic effects associated with the substituent on the phenyl ring. In particular, *para* substitution may influence both the preferred mol­ecular conformation and the inter­molecular packing arrangement. Thus, the structural features observed for compound **3**, including the planarity of the heteroaromatic core, the orientation of the benzoyl phenyl ring and the presence of *π–π* inter­actions, are broadly consistent with the structural trends evident from related thia­zolo[3,2-*a*]benzimidazole derivatives reported in the CSD.

## 5. Synthesis and crystallization

A mixture of 1.0 equiv of 2-mercaptobenzimidazole (**1**), 1.0 equiv of (*E*)-1-(4-chloro­phen­yl)-3-(di­methyl­amino)­prop-2-en-1-one (**2**), and 1.0 equiv of iodine in acetic acid (5 mL) was stirred at room temperature under open air for 2 h (Fig. 7 ▸). Upon completion (monitored by TLC), the reaction mixture was poured into water, quenched with aqueous sodium thio­sulfate, and extracted with ethyl acetate. The combined organic layers were dried over Na_2_SO_4_ and concentrated under reduced pressure. The crude product, (benzo[4,5]imidazo[2,1-*b*]thia­zol-2-yl)(4-chloro­phen­yl)methanone (**3**) was purified by column chromatography and the well dried sample was crystallized using 100% ethyl­acetate as a solvent by slow evaporation method which yielded pale yellow crystal for diffraction study.

## 6. Refinement

Crystal data, data collection and structure refinement details are summarized in Table 3 ▸. The carbon-bound hydrogen atoms were positioned based on calculated values (C—H = 0.93 Å) and were refined as riding atoms, with *U*_iso_*(H)* set to 1.2*U*_eq_*(C)*.

## Supplementary Material

Crystal structure: contains datablock(s) I. DOI: 10.1107/S2056989026010091/ox2026sup1.cif

Structure factors: contains datablock(s) I. DOI: 10.1107/S2056989026010091/ox2026Isup2.hkl

Figure S1A. Hirshfeld surface fingerprint plots for Molecule A. The full plot (all...all = 100.0%) and delineated specific atomic contacts are shown with different quantity. Blue highlighted areas represent the respective interactions, with percentage contributions labeled. DOI: 10.1107/S2056989026010091/ox2026sup3.tif

Figure S1B. Hirshfeld surface fingerprint plots for Molecule B. The full plot (all...all = 100.0%) and delineated specific atomic contacts are shown with different quantity. Blue highlighted areas represent the respective interactions, with percentage contributions labeled. DOI: 10.1107/S2056989026010091/ox2026sup4.tif

Supporting information file. DOI: 10.1107/S2056989026010091/ox2026Isup5.cml

CCDC reference: 2591449

Additional supporting information:  crystallographic information; 3D view; checkCIF report

## Figures and Tables

**Figure 1:**
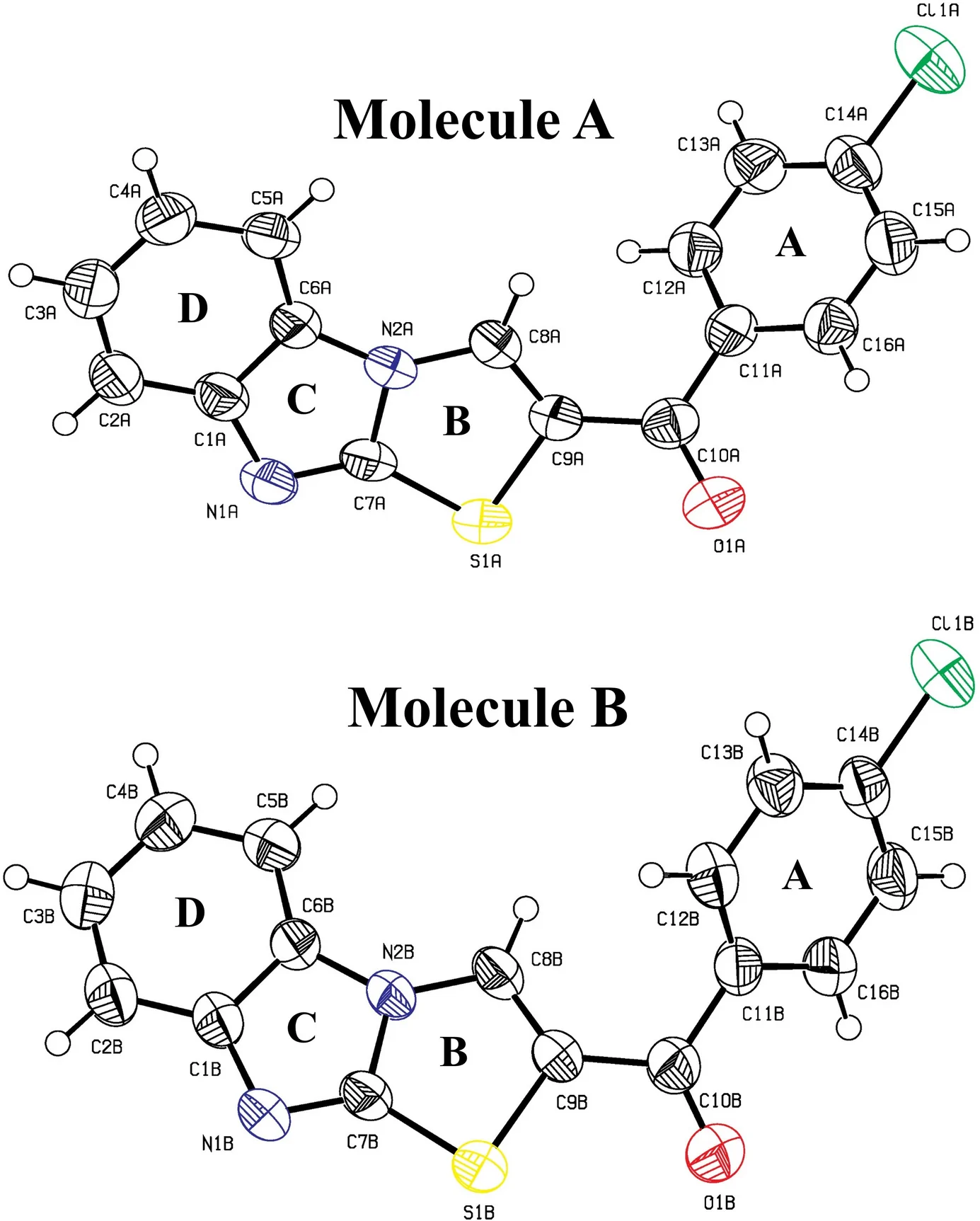
The mol­ecular structures (mol­ecules *A* and *B*) of compound **3** showing 50% displacement ellipsoids.

**Figure 2:**
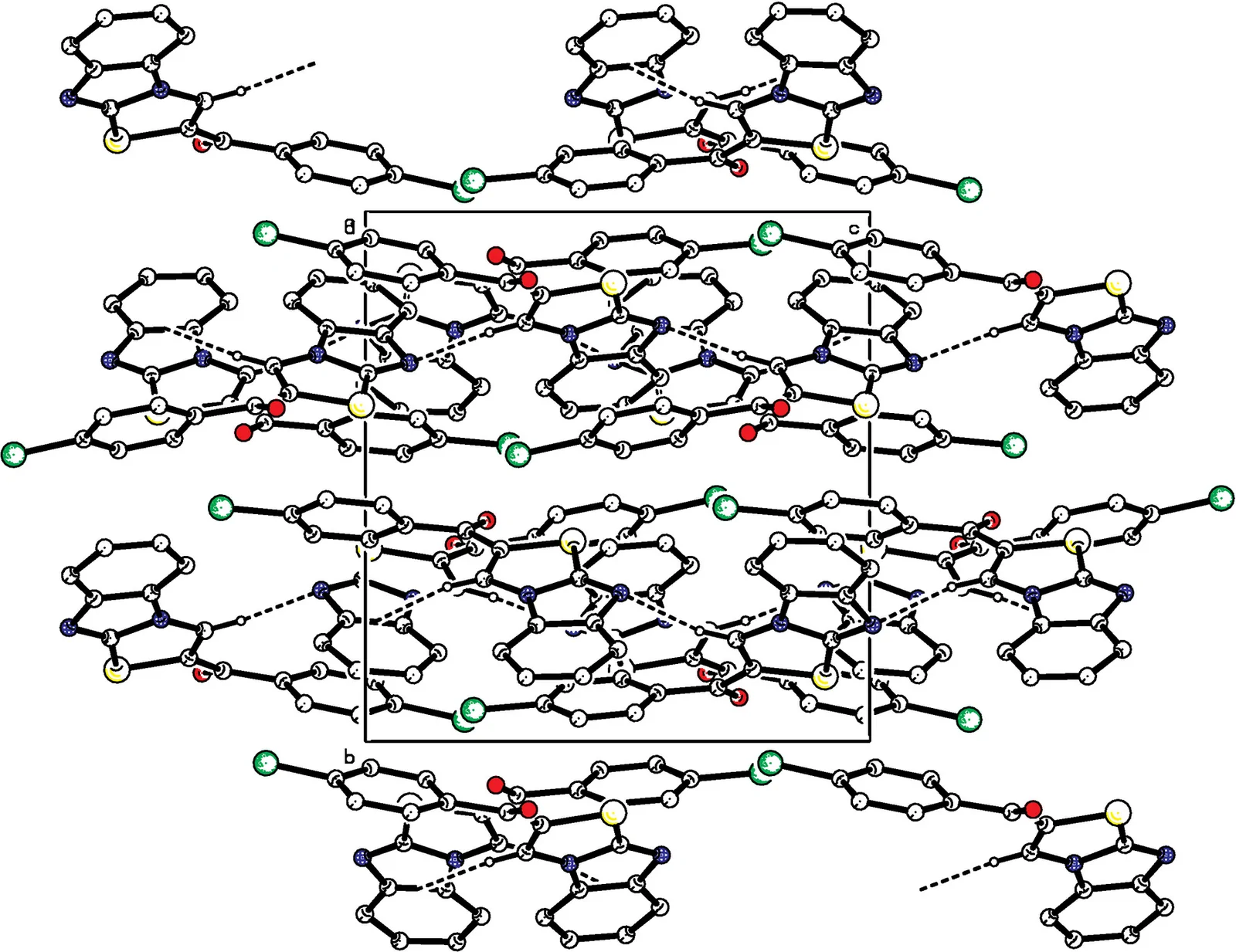
Packing diagram for compound **3** viewed down the *a* axis in the crystal unit cell. The dotted lines indicate the hydrogen bonds between symmetry-related mol­ecules, which extend as C—H⋯N inter­molecular inter­actions towards the *c-*axis direction in the unit-cell packing.

**Figure 3:**
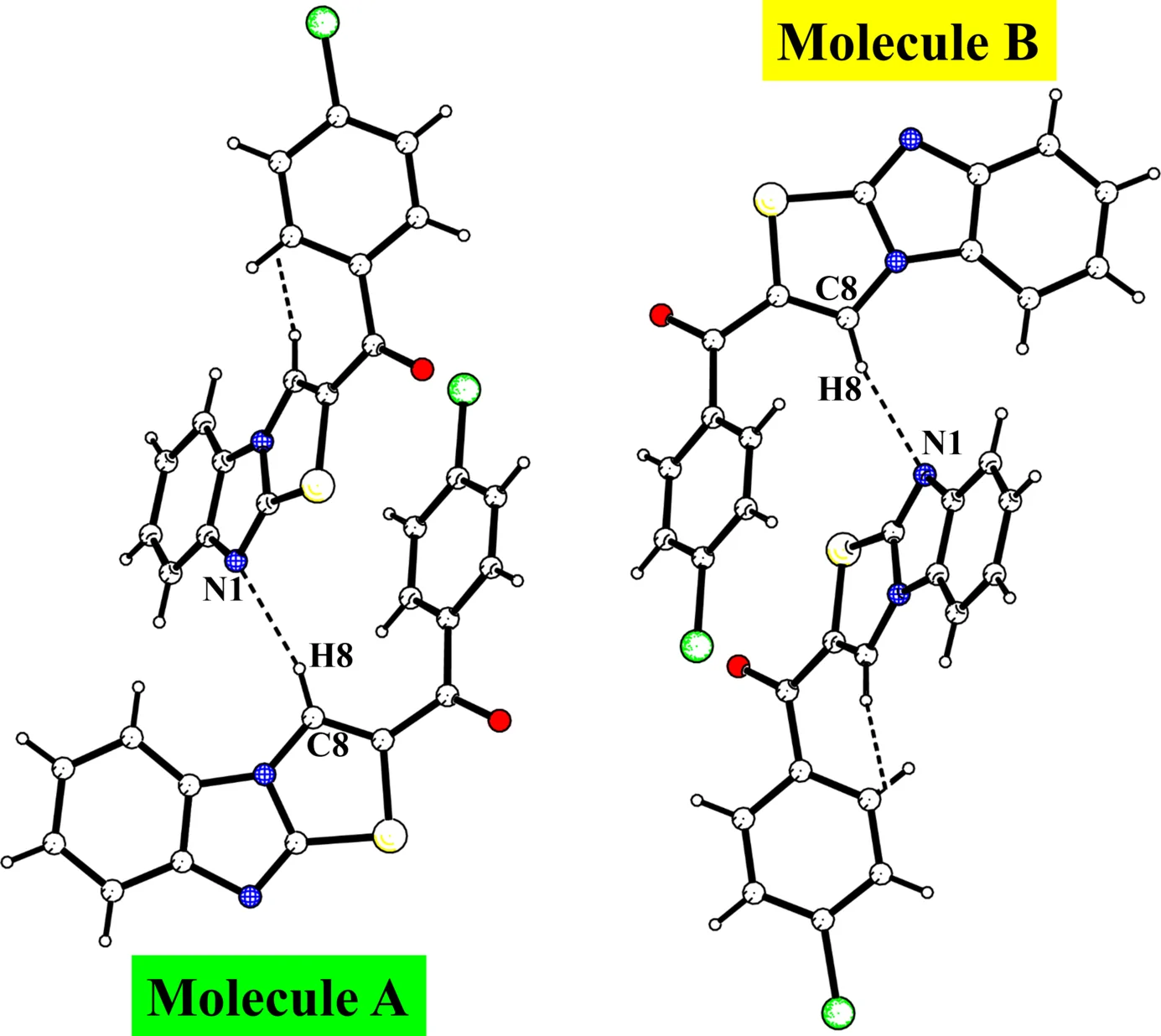
Hydrogen-bonding inter­actions in the crystal structure of compound **3**, showing the corresponding inter­molecular contacts (C—H⋯N) involving the two crystallographically independent mol­ecules ***A*** and *B*. Dashed lines indicate the hydrogen-bond inter­actions.

**Figure 4:**
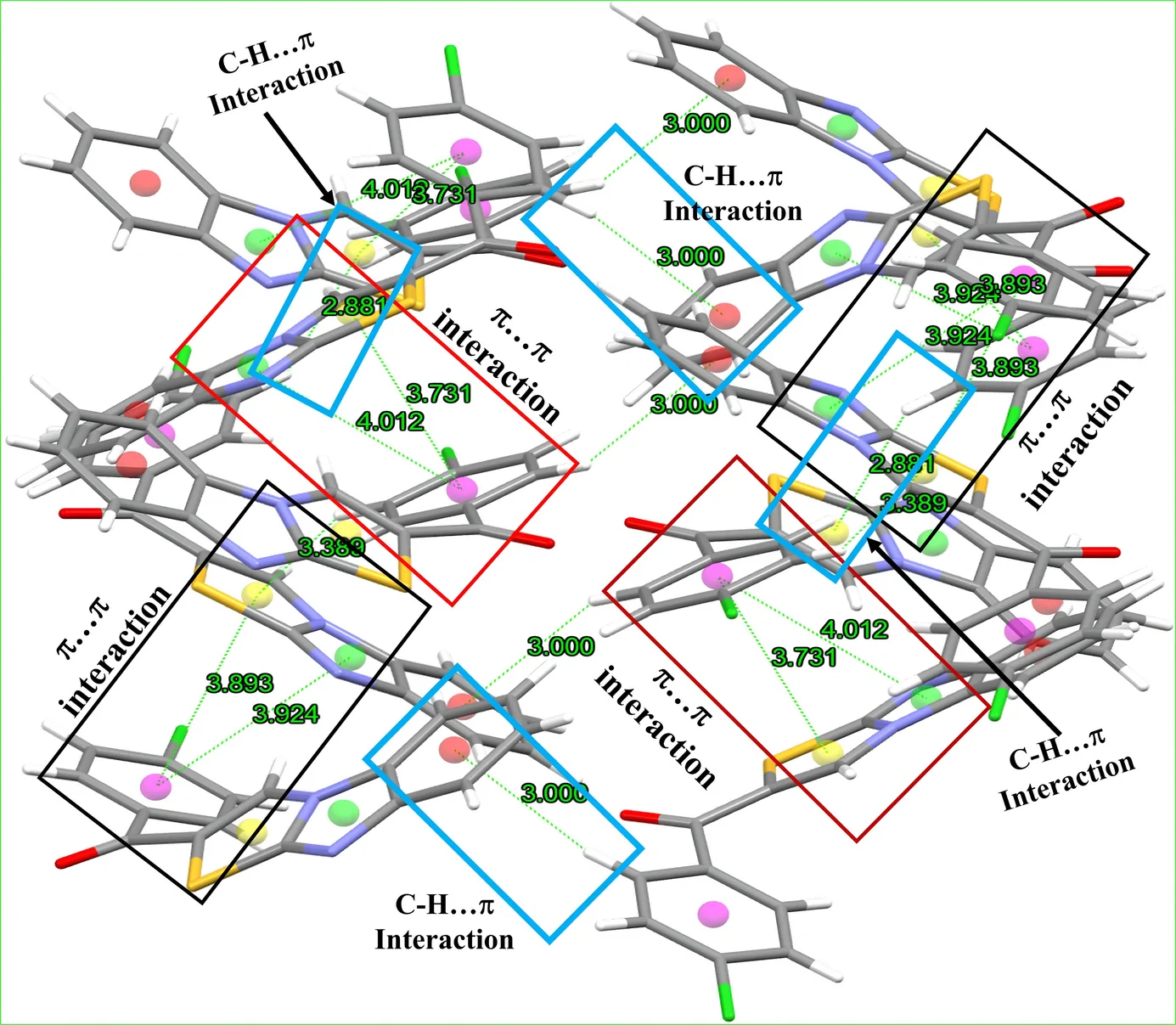
Visualization of inter­molecular C—H⋯π (blue boxes) and π–π (red and black boxes) inter­actions in the crystal of compound **3**. Green dashed lines indicate inter­action distances (Å).

**Figure 5:**
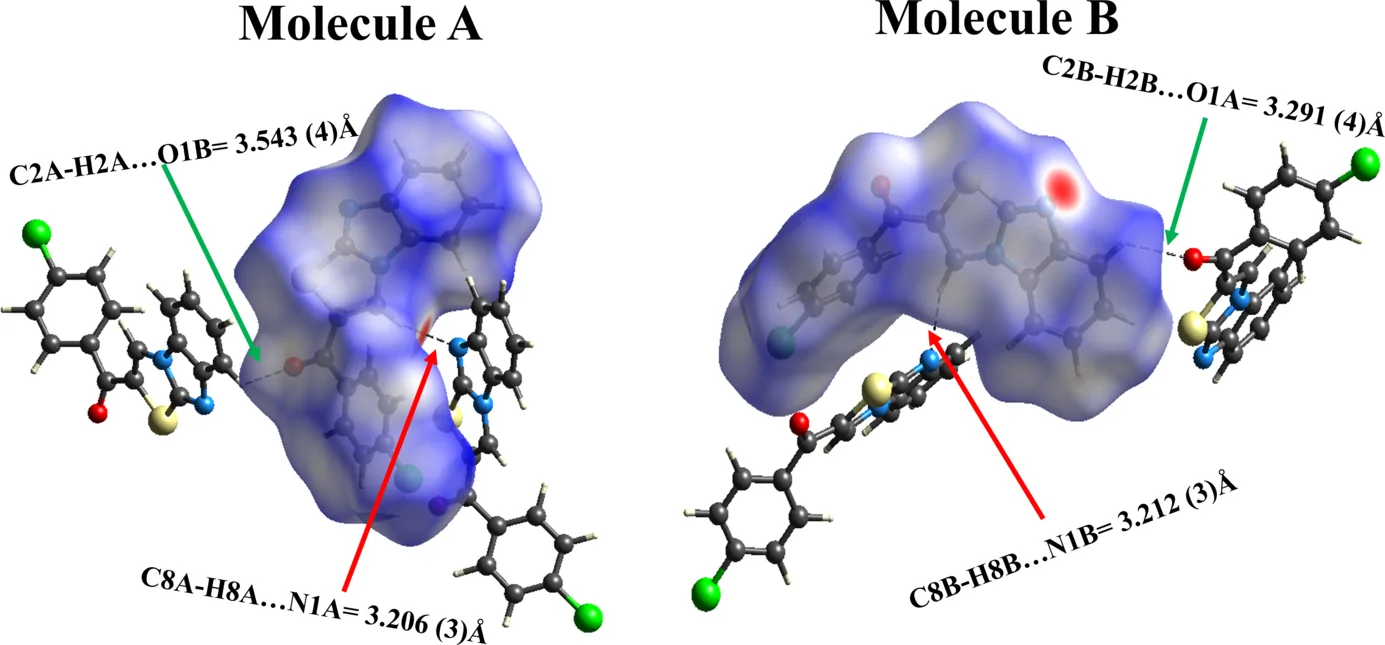
Hirshfeld surface mapped with *d*_norm_ for compound **3**. Both mol­ecules *A* and *B* form two types of inter­molecular inter­actions, N—H⋯O and C—H⋯O, which contribute significantly to the cohesion of the crystal.

**Figure 6:**
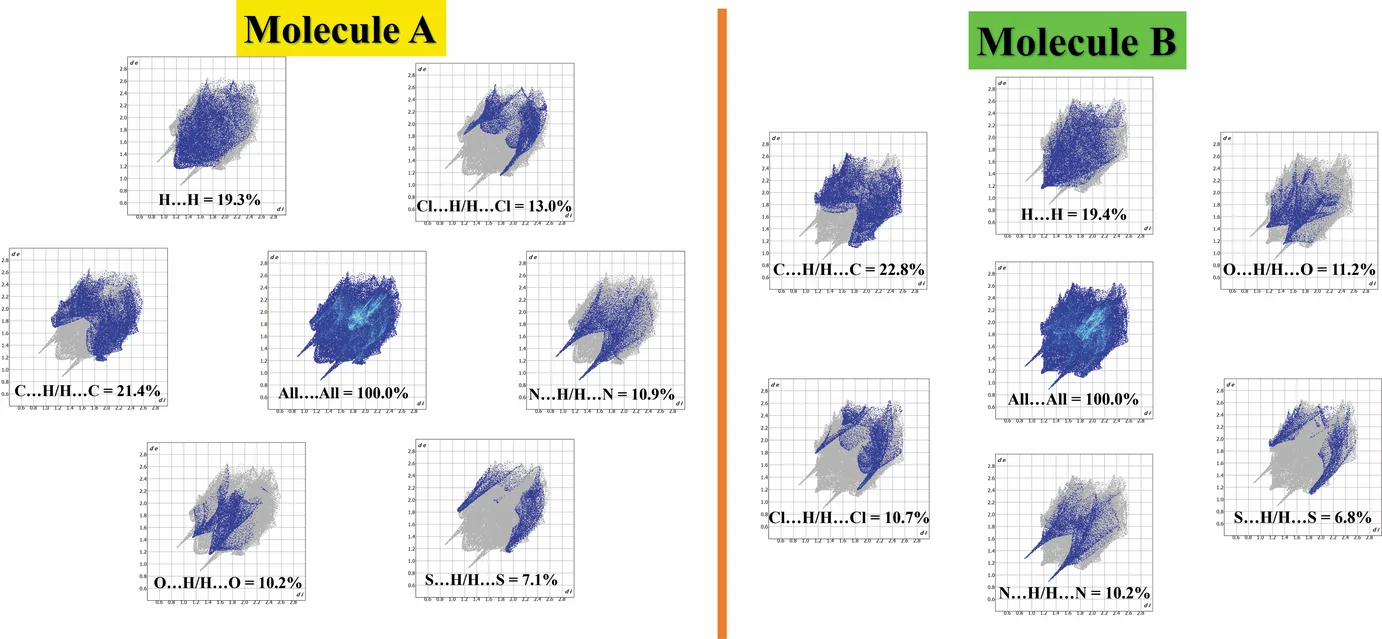
Hirshfeld surface fingerprint plots for compound **3** for mol­ecules *A* and *B*. Contacts contributing >5% to the total surface are given with their respective percentage contributions labeled; those contributing less than 5% are shown in supplementary Figs. S1*A* and S1*B*.

**Figure 7:**
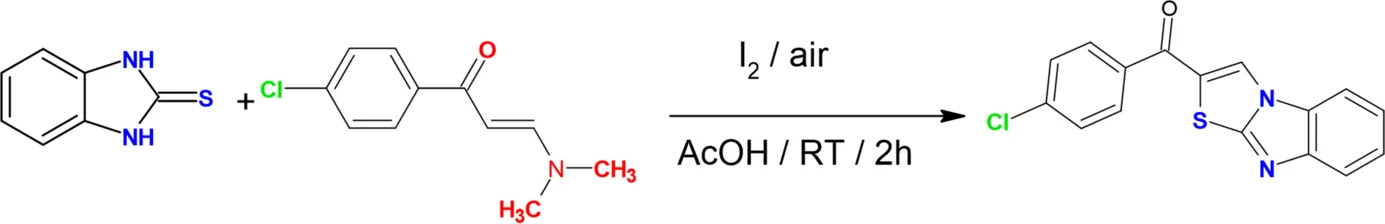
Synthetic route of the title compound (**3**).

**Table 1:** Selected geometric parameters (Å, °)

S1*B*—C7*B*	1.725 (3)	N2*A*—C8*A*	1.375 (3)
S1*B*—C9*B*	1.760 (3)	N2*A*—C6*A*	1.390 (3)
S1*A*—C7*A*	1.726 (3)	O1*A*—C10*A*	1.220 (3)
S1*A*—C9*A*	1.762 (3)	N1*B*—C7*B*	1.305 (3)
N2*B*—C7*B*	1.382 (3)	N1*B*—C1*B*	1.404 (4)
N2*B*—C8*B*	1.370 (3)	O1*B*—C10*B*	1.219 (3)
N2*B*—C6*B*	1.393 (3)	N1*A*—C7*A*	1.305 (3)
N2*A*—C7*A*	1.383 (3)	N1*A*—C1*A*	1.407 (4)
			
C7*B*—S1*B*—C9*B*	89.83 (13)	N1*B*—C7*B*—N2*B*	114.3 (2)
C7*A*—S1*A*—C9*A*	89.93 (14)	C9*B*—C8*B*—N2*B*	112.2 (2)
C7*B*—N2*B*—C6*B*	106.8 (2)	C9*A*—C8*A*—N2*A*	112.7 (3)
C8*B*—N2*B*—C7*B*	114.8 (2)	C8*B*—C9*B*—S1*B*	112.4 (2)
C8*B*—N2*B*—C6*B*	138.4 (2)	C10*B*—C9*B*—S1*B*	120.1 (2)
C7*A*—N2*A*—C6*A*	106.7 (2)	N1*B*—C1*B*—C6*B*	111.8 (2)
C8*A*—N2*A*—C7*A*	114.4 (2)	C2*B*—C1*B*—N1*B*	129.5 (3)
C8*A*—N2*A*—C6*A*	138.9 (2)	C8*A*—C9*A*—S1*A*	112.2 (2)
C7*B*—N1*B*—C1*B*	103.4 (2)	C10*A*—C9*A*—S1*A*	119.9 (2)
C7*A*—N1*A*—C1*A*	103.4 (2)	N2*B*—C6*B*—C1*B*	103.8 (2)
N2*A*—C7*A*—S1*A*	110.8 (2)	C5*B*—C6*B*—N2*B*	132.5 (3)
N1*A*—C7*A*—S1*A*	135.0 (2)	N2*A*—C6*A*—C1*A*	104.1 (2)
N1*A*—C7*A*—N2*A*	114.2 (3)	C5*A*—C6*A*—N2*A*	132.0 (3)
N2*B*—C7*B*—S1*B*	110.7 (2)	C6*A*—C1*A*—N1*A*	111.6 (3)
N1*B*—C7*B*—S1*B*	135.0 (2)	C2*A*—C1*A*—N1*A*	129.8 (3)

**Table 2:** Hydrogen-bond geometry (Å, °) *Cg*1, and *Cg*7 are the centroids of of atoms S1*A*/C7*A*/N2*A*/C8*A*/C9*A* and C1*B*–C6*B*, respectively.

*D*—H⋯*A*	*D*—H	H⋯*A*	*D*⋯*A*	*D*—H⋯*A*
C8*A*—H8*A*⋯N1*A*^i^	0.93	2.30	3.206 (3)	165
C8*B*—H8*B*⋯N1*B*^ii^	0.93	2.31	3.212 (3)	163
C13*B*—H13*B*⋯*Cg*1^ii^	0.93	2.88	3.551 (4)	130
C16*A*—H16*A*⋯*Cg*7^iii^	0.93	3.00	3.832 (4)	150

Symmetry codes: (i) 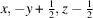; (ii) 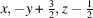; (iii) 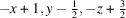.

**Table 3:** Experimental details

Crystal data	
Chemical formula	C_16_H_9_ClN_2_OS
*M*_r_	312.76
Crystal system, space group	Monoclinic, *P*2_1_/*c*
Temperature (K)	300
*a*, *b*, *c* (Å)	18.1878 (18), 12.8058 (13), 12.1917 (11)
β (°)	90.115 (4)
*V* (Å^3^)	2839.6 (5)
*Z*	8
Radiation type	Mo *K*α
μ (mm^−1^)	0.42
Crystal size (mm)	0.30 × 0.15 × 0.10

Data collection	
Diffractometer	Bruker APEXII CCD
Absorption correction	Multi-scan (*SADABS*; Krause *et al.*, 2015 ▸)
*T*_min_, *T*_max_	0.678, 0.746
No. of measured, independent and observed [*I* > 2σ(*I*)] reflections	94667, 7077, 3706
*R*_int_	0.096
(sin θ/λ)_max_ (Å^−1^)	0.668

Refinement	
*R*[*F*^2^ > 2σ(*F*^2^)], *wR*(*F*^2^), *S*	0.059, 0.134, 1.11
No. of reflections	7077
No. of parameters	380
H-atom treatment	H-atom parameters constrained
Δρ_max_, Δρ_min_ (e Å^−3^)	0.38, −0.33

Computer programs: *APEX3* (Bruker, 2016 ▸), *SAINT* (Bruker, 2017 ▸), *OLEX2.solve* (Bourhis *et al.*, 2015 ▸), *SHELXL2019/3* (Sheldrick, 2015 ▸) and *OLEX2* (Dolomanov *et al.*, 2009 ▸).

## supplementary crystallographic information

### (Benzo[4,5]imidazo[2,1-b]thiazol-2-yl)(4-chlorophenyl)methanone Crystal data

**Table:**

C_16_H_9_ClN_2_OS	*F*(000) = 1280
*M**_r_* = 312.76	*D*_x_ = 1.463 Mg m^−^^3^
Monoclinic, *P*2_1_/*c*	Mo *K*α radiation, λ = 0.71073 Å
*a* = 18.1878 (18) Å	Cell parameters from 94667 reflections
*b* = 12.8058 (13) Å	θ = 2.0–28.4°
*c* = 12.1917 (11) Å	µ = 0.42 mm^−^^1^
β = 90.115 (4)°	*T* = 300 K
*V* = 2839.6 (5) Å^3^	Block, pale yellow
*Z* = 8	0.30 × 0.15 × 0.10 mm

### (Benzo[4,5]imidazo[2,1-b]thiazol-2-yl)(4-chlorophenyl)methanone Data collection

**Table:**

Bruker APEXII CCD diffractometer	7077 independent reflections
Radiation source: MoKα (0.71073)	3706 reflections with *I* > 2σ(*I*)
Graphite monochromator	*R*_int_ = 0.096
φ and ω scans	θ_max_ = 28.4°, θ_min_ = 2.0°
Absorption correction: multi-scan (SADABS; Krause *et al.*, 2015)	*h* = −24→24
*T*_min_ = 0.678, *T*_max_ = 0.746	*k* = −17→17
94667 measured reflections	*l* = −16→16

### (Benzo[4,5]imidazo[2,1-b]thiazol-2-yl)(4-chlorophenyl)methanone Refinement

**Table:**

Refinement on *F*^2^	Primary atom site location: iterative
Least-squares matrix: full	Hydrogen site location: inferred from neighbouring sites
*R*[*F*^2^ > 2σ(*F*^2^)] = 0.059	H-atom parameters constrained
*wR*(*F*^2^) = 0.134	*w* = 1/[σ^2^(*F*_o_^2^) + (0.0264*P*)^2^ + 2.3482*P*] where *P* = (*F*_o_^2^ + 2*F*_c_^2^)/3
*S* = 1.11	(Δ/σ)_max_ < 0.001
7077 reflections	Δρ_max_ = 0.38 e Å^−^^3^
380 parameters	Δρ_min_ = −0.33 e Å^−^^3^
0 restraints	Extinction correction: SHELXL2019/2 (Sheldrick 2015), Fc^*^=kFc[1+0.001xFc^2^λ^3^/sin(2θ)]^-1/4^
0 constraints	Extinction coefficient: 0.0031 (3)

### (Benzo[4,5]imidazo[2,1-b]thiazol-2-yl)(4-chlorophenyl)methanone Special details

**Table:**

Geometry. All esds (except the esd in the dihedral angle between two l.s. planes) are estimated using the full covariance matrix. The cell esds are taken into account individually in the estimation of esds in distances, angles and torsion angles; correlations between esds in cell parameters are only used when they are defined by crystal symmetry. An approximate (isotropic) treatment of cell esds is used for estimating esds involving l.s. planes.

### (Benzo[4,5]imidazo[2,1-b]thiazol-2-yl)(4-chlorophenyl)methanone Fractional atomic coordinates and isotropic or equivalent isotropic displacement parameters (Å^2^)

**Table:**

	*x*	*y*	*z*	*U*_iso_*/*U*_eq_	
S1B	0.15689 (4)	0.87765 (7)	0.91119 (6)	0.0579 (3)	
S1A	0.34650 (4)	0.36471 (7)	0.99192 (6)	0.0572 (2)	
Cl1B	0.13842 (6)	0.94112 (9)	0.21428 (7)	0.0852 (3)	
Cl1A	0.37444 (7)	0.45888 (10)	0.30319 (8)	0.0999 (4)	
N2B	0.26281 (12)	0.77668 (19)	0.82212 (16)	0.0444 (6)	
N2A	0.23686 (13)	0.2692 (2)	0.90499 (17)	0.0474 (6)	
O1A	0.46914 (12)	0.3712 (2)	0.82364 (18)	0.0708 (7)	
N1B	0.27979 (13)	0.7804 (2)	1.00557 (17)	0.0505 (6)	
O1B	0.03999 (13)	0.9172 (2)	0.74099 (18)	0.0804 (8)	
N1A	0.21787 (14)	0.2853 (2)	1.08714 (18)	0.0532 (7)	
C7A	0.26191 (16)	0.3049 (2)	1.0054 (2)	0.0480 (7)	
C7B	0.23775 (15)	0.8083 (2)	0.9238 (2)	0.0466 (7)	
C8B	0.21990 (16)	0.8074 (2)	0.7354 (2)	0.0496 (8)	
H8B	0.230338	0.791704	0.662595	0.060*	
C8A	0.28273 (16)	0.2910 (2)	0.8184 (2)	0.0492 (8)	
H8A	0.272567	0.272234	0.746268	0.059*	
C9B	0.16081 (16)	0.8629 (2)	0.7678 (2)	0.0483 (7)	
C1B	0.33663 (16)	0.7245 (2)	0.9545 (2)	0.0482 (7)	
C9A	0.34387 (16)	0.3422 (2)	0.8494 (2)	0.0496 (8)	
C6B	0.32728 (15)	0.7205 (2)	0.8401 (2)	0.0455 (7)	
C5A	0.12219 (17)	0.1675 (3)	0.8555 (2)	0.0548 (8)	
H5A	0.130929	0.160840	0.780738	0.066*	
C6A	0.17003 (16)	0.2200 (2)	0.9242 (2)	0.0474 (7)	
C10B	0.09936 (17)	0.8982 (3)	0.6993 (2)	0.0540 (8)	
C1A	0.15944 (16)	0.2308 (2)	1.0375 (2)	0.0499 (8)	
C11B	0.11086 (16)	0.9082 (2)	0.5789 (2)	0.0498 (8)	
C5B	0.37464 (17)	0.6696 (3)	0.7704 (2)	0.0554 (8)	
H5B	0.366915	0.667852	0.694953	0.066*	
C11A	0.39723 (16)	0.3855 (2)	0.6625 (2)	0.0482 (7)	
C3B	0.44454 (18)	0.6231 (3)	0.9315 (3)	0.0605 (9)	
H3B	0.485091	0.588914	0.961101	0.073*	
C2A	0.09724 (17)	0.1867 (3)	1.0844 (3)	0.0578 (8)	
H2A	0.088412	0.192151	1.159327	0.069*	
C2B	0.39681 (17)	0.6737 (3)	1.0008 (3)	0.0574 (8)	
H2B	0.404510	0.674055	1.076267	0.069*	
C10A	0.40817 (17)	0.3665 (2)	0.7820 (2)	0.0524 (8)	
C4B	0.43413 (17)	0.6213 (3)	0.8186 (3)	0.0590 (8)	
H4B	0.468062	0.586749	0.774676	0.071*	
C4A	0.06062 (17)	0.1256 (3)	0.9047 (3)	0.0612 (9)	
H4A	0.026178	0.090667	0.861837	0.073*	
C16A	0.45472 (17)	0.3662 (3)	0.5907 (3)	0.0582 (8)	
H16A	0.498579	0.338939	0.617570	0.070*	
C3A	0.04902 (18)	0.1345 (3)	1.0165 (3)	0.0620 (9)	
H3A	0.007262	0.104148	1.046830	0.074*	
C16B	0.05378 (17)	0.8820 (3)	0.5086 (2)	0.0589 (9)	
H16B	0.009540	0.857925	0.537172	0.071*	
C14B	0.12725 (19)	0.9288 (3)	0.3553 (2)	0.0562 (8)	
C12B	0.17583 (18)	0.9459 (3)	0.5350 (3)	0.0584 (8)	
H12B	0.214242	0.964260	0.581668	0.070*	
C13B	0.18442 (18)	0.9566 (3)	0.4230 (3)	0.0597 (9)	
H13B	0.228124	0.982144	0.394028	0.072*	
C12A	0.33295 (17)	0.4283 (3)	0.6207 (3)	0.0567 (8)	
H12A	0.294090	0.442577	0.667799	0.068*	
C15B	0.06211 (18)	0.8912 (3)	0.3964 (3)	0.0635 (9)	
H15B	0.024128	0.872187	0.349270	0.076*	
C14A	0.3837 (2)	0.4293 (3)	0.4417 (3)	0.0624 (9)	
C13A	0.32574 (19)	0.4500 (3)	0.5107 (3)	0.0613 (9)	
H13A	0.282300	0.478202	0.483318	0.074*	
C15A	0.44796 (19)	0.3868 (3)	0.4805 (3)	0.0684 (10)	
H15A	0.486426	0.372046	0.432761	0.082*	

### (Benzo[4,5]imidazo[2,1-b]thiazol-2-yl)(4-chlorophenyl)methanone Atomic displacement parameters (Å^2^)

**Table:**

	*U*^11^	*U*^22^	*U*^33^	*U*^12^	*U*^13^	*U*^23^
S1B	0.0545 (5)	0.0800 (6)	0.0391 (4)	0.0107 (4)	0.0005 (3)	−0.0064 (4)
S1A	0.0545 (5)	0.0729 (6)	0.0441 (4)	−0.0056 (4)	−0.0096 (3)	−0.0040 (4)
Cl1B	0.1001 (8)	0.1075 (9)	0.0479 (5)	0.0073 (6)	−0.0047 (5)	0.0176 (5)
Cl1A	0.1188 (9)	0.1223 (10)	0.0586 (6)	−0.0032 (7)	0.0062 (6)	0.0263 (6)
N2B	0.0497 (14)	0.0530 (16)	0.0304 (11)	0.0036 (12)	−0.0023 (10)	−0.0024 (11)
N2A	0.0508 (15)	0.0585 (17)	0.0329 (12)	−0.0029 (12)	−0.0046 (10)	0.0018 (11)
O1A	0.0494 (13)	0.0950 (19)	0.0679 (15)	−0.0010 (13)	−0.0091 (11)	0.0039 (13)
N1B	0.0559 (15)	0.0625 (18)	0.0333 (12)	0.0021 (13)	−0.0019 (11)	0.0010 (12)
O1B	0.0607 (15)	0.125 (2)	0.0555 (14)	0.0245 (15)	−0.0010 (12)	−0.0075 (14)
N1A	0.0583 (16)	0.0636 (18)	0.0375 (13)	0.0015 (14)	−0.0052 (12)	0.0008 (12)
C7A	0.0531 (18)	0.055 (2)	0.0359 (15)	0.0030 (15)	−0.0086 (13)	0.0006 (14)
C7B	0.0499 (17)	0.057 (2)	0.0328 (14)	−0.0007 (15)	−0.0001 (12)	−0.0027 (14)
C8B	0.0564 (18)	0.061 (2)	0.0316 (14)	−0.0004 (16)	−0.0038 (13)	0.0019 (14)
C8A	0.0535 (18)	0.056 (2)	0.0380 (15)	−0.0022 (16)	0.0008 (13)	0.0014 (14)
C9B	0.0508 (17)	0.059 (2)	0.0351 (14)	0.0021 (16)	−0.0033 (13)	−0.0024 (14)
C1B	0.0518 (18)	0.051 (2)	0.0413 (16)	−0.0027 (15)	−0.0057 (13)	0.0030 (14)
C9A	0.0480 (18)	0.060 (2)	0.0402 (15)	0.0002 (15)	−0.0033 (13)	0.0003 (14)
C6B	0.0435 (16)	0.0521 (19)	0.0408 (15)	−0.0006 (15)	−0.0011 (12)	0.0050 (14)
C5A	0.059 (2)	0.062 (2)	0.0436 (16)	−0.0039 (17)	−0.0080 (14)	0.0015 (15)
C6A	0.0480 (17)	0.052 (2)	0.0418 (16)	−0.0041 (15)	−0.0020 (13)	0.0052 (14)
C10B	0.0536 (19)	0.063 (2)	0.0456 (17)	0.0052 (17)	−0.0051 (14)	−0.0062 (15)
C1A	0.0543 (19)	0.054 (2)	0.0416 (16)	0.0054 (16)	−0.0016 (14)	0.0051 (14)
C11B	0.0508 (18)	0.053 (2)	0.0458 (16)	0.0089 (15)	−0.0082 (14)	0.0001 (14)
C5B	0.060 (2)	0.062 (2)	0.0443 (16)	0.0053 (17)	0.0046 (15)	0.0020 (16)
C11A	0.0459 (17)	0.0456 (19)	0.0532 (17)	−0.0021 (14)	0.0010 (14)	−0.0008 (15)
C3B	0.0536 (19)	0.056 (2)	0.072 (2)	0.0004 (17)	−0.0110 (17)	0.0080 (18)
C2A	0.061 (2)	0.066 (2)	0.0459 (17)	0.0057 (18)	0.0063 (15)	0.0067 (16)
C2B	0.060 (2)	0.063 (2)	0.0490 (17)	−0.0053 (17)	−0.0116 (15)	0.0064 (16)
C10A	0.0501 (19)	0.052 (2)	0.0550 (18)	0.0032 (16)	−0.0030 (15)	−0.0015 (16)
C4B	0.0532 (19)	0.057 (2)	0.067 (2)	0.0048 (17)	0.0034 (16)	−0.0012 (17)
C4A	0.055 (2)	0.065 (2)	0.064 (2)	−0.0047 (18)	−0.0072 (16)	0.0036 (18)
C16A	0.0518 (19)	0.062 (2)	0.060 (2)	0.0036 (17)	0.0056 (15)	0.0036 (17)
C3A	0.0540 (19)	0.064 (2)	0.068 (2)	0.0002 (18)	0.0056 (17)	0.0080 (19)
C16B	0.0507 (18)	0.074 (2)	0.0519 (18)	0.0000 (17)	−0.0050 (15)	0.0032 (17)
C14B	0.066 (2)	0.056 (2)	0.0466 (17)	0.0067 (17)	−0.0092 (15)	0.0073 (16)
C12B	0.062 (2)	0.061 (2)	0.0527 (19)	−0.0036 (17)	−0.0145 (15)	0.0049 (16)
C13B	0.061 (2)	0.060 (2)	0.058 (2)	−0.0060 (17)	−0.0053 (16)	0.0117 (17)
C12A	0.0518 (19)	0.062 (2)	0.0559 (19)	0.0025 (16)	0.0046 (15)	0.0040 (17)
C15B	0.059 (2)	0.079 (3)	0.0525 (19)	−0.0017 (19)	−0.0197 (16)	0.0015 (18)
C14A	0.074 (2)	0.064 (2)	0.0491 (18)	−0.0061 (19)	0.0062 (17)	0.0084 (17)
C13A	0.063 (2)	0.057 (2)	0.064 (2)	0.0017 (17)	−0.0048 (17)	0.0090 (17)
C15A	0.065 (2)	0.079 (3)	0.061 (2)	0.000 (2)	0.0168 (17)	0.0056 (19)

### (Benzo[4,5]imidazo[2,1-b]thiazol-2-yl)(4-chlorophenyl)methanone Geometric parameters (Å, º)

**Table:**

S1B—C7B	1.725 (3)	C11B—C16B	1.386 (4)
S1B—C9B	1.760 (3)	C11B—C12B	1.385 (4)
S1A—C7A	1.726 (3)	C5B—H5B	0.9300
S1A—C9A	1.762 (3)	C5B—C4B	1.377 (4)
Cl1B—C14B	1.739 (3)	C11A—C10A	1.491 (4)
Cl1A—C14A	1.739 (3)	C11A—C16A	1.387 (4)
N2B—C7B	1.382 (3)	C11A—C12A	1.387 (4)
N2B—C8B	1.370 (3)	C3B—H3B	0.9300
N2B—C6B	1.393 (3)	C3B—C2B	1.376 (4)
N2A—C7A	1.383 (3)	C3B—C4B	1.389 (4)
N2A—C8A	1.375 (3)	C2A—H2A	0.9300
N2A—C6A	1.390 (3)	C2A—C3A	1.378 (4)
O1A—C10A	1.220 (3)	C2B—H2B	0.9300
N1B—C7B	1.305 (3)	C4B—H4B	0.9300
N1B—C1B	1.404 (4)	C4A—H4A	0.9300
O1B—C10B	1.219 (3)	C4A—C3A	1.384 (4)
N1A—C7A	1.305 (3)	C16A—H16A	0.9300
N1A—C1A	1.407 (4)	C16A—C15A	1.375 (4)
C8B—H8B	0.9300	C3A—H3A	0.9300
C8B—C9B	1.349 (4)	C16B—H16B	0.9300
C8A—H8A	0.9300	C16B—C15B	1.381 (4)
C8A—C9A	1.345 (4)	C14B—C13B	1.373 (4)
C9B—C10B	1.466 (4)	C14B—C15B	1.374 (4)
C1B—C6B	1.405 (4)	C12B—H12B	0.9300
C1B—C2B	1.392 (4)	C12B—C13B	1.382 (4)
C9A—C10A	1.464 (4)	C13B—H13B	0.9300
C6B—C5B	1.376 (4)	C12A—H12A	0.9300
C5A—H5A	0.9300	C12A—C13A	1.375 (4)
C5A—C6A	1.381 (4)	C15B—H15B	0.9300
C5A—C4A	1.380 (4)	C14A—C13A	1.375 (4)
C6A—C1A	1.402 (4)	C14A—C15A	1.372 (5)
C10B—C11B	1.488 (4)	C13A—H13A	0.9300
C1A—C2A	1.389 (4)	C15A—H15A	0.9300
			
C7B—S1B—C9B	89.83 (13)	C16A—C11A—C12A	118.3 (3)
C7A—S1A—C9A	89.93 (14)	C12A—C11A—C10A	122.3 (3)
C7B—N2B—C6B	106.8 (2)	C2B—C3B—H3B	118.9
C8B—N2B—C7B	114.8 (2)	C2B—C3B—C4B	122.1 (3)
C8B—N2B—C6B	138.4 (2)	C4B—C3B—H3B	118.9
C7A—N2A—C6A	106.7 (2)	C1A—C2A—H2A	121.0
C8A—N2A—C7A	114.4 (2)	C3A—C2A—C1A	117.9 (3)
C8A—N2A—C6A	138.9 (2)	C3A—C2A—H2A	121.0
C7B—N1B—C1B	103.4 (2)	C1B—C2B—H2B	121.1
C7A—N1A—C1A	103.4 (2)	C3B—C2B—C1B	117.8 (3)
N2A—C7A—S1A	110.8 (2)	C3B—C2B—H2B	121.1
N1A—C7A—S1A	135.0 (2)	O1A—C10A—C9A	120.2 (3)
N1A—C7A—N2A	114.2 (3)	O1A—C10A—C11A	121.2 (3)
N2B—C7B—S1B	110.7 (2)	C9A—C10A—C11A	118.6 (3)
N1B—C7B—S1B	135.0 (2)	C5B—C4B—C3B	121.4 (3)
N1B—C7B—N2B	114.3 (2)	C5B—C4B—H4B	119.3
N2B—C8B—H8B	123.9	C3B—C4B—H4B	119.3
C9B—C8B—N2B	112.2 (2)	C5A—C4A—H4A	119.3
C9B—C8B—H8B	123.9	C5A—C4A—C3A	121.4 (3)
N2A—C8A—H8A	123.7	C3A—C4A—H4A	119.3
C9A—C8A—N2A	112.7 (3)	C11A—C16A—H16A	119.4
C9A—C8A—H8A	123.7	C15A—C16A—C11A	121.1 (3)
C8B—C9B—S1B	112.4 (2)	C15A—C16A—H16A	119.4
C8B—C9B—C10B	127.1 (3)	C2A—C3A—C4A	122.2 (3)
C10B—C9B—S1B	120.1 (2)	C2A—C3A—H3A	118.9
N1B—C1B—C6B	111.8 (2)	C4A—C3A—H3A	118.9
C2B—C1B—N1B	129.5 (3)	C11B—C16B—H16B	119.7
C2B—C1B—C6B	118.7 (3)	C15B—C16B—C11B	120.5 (3)
C8A—C9A—S1A	112.2 (2)	C15B—C16B—H16B	119.7
C8A—C9A—C10A	127.4 (3)	C13B—C14B—Cl1B	118.8 (3)
C10A—C9A—S1A	119.9 (2)	C13B—C14B—C15B	121.6 (3)
N2B—C6B—C1B	103.8 (2)	C15B—C14B—Cl1B	119.6 (2)
C5B—C6B—N2B	132.5 (3)	C11B—C12B—H12B	119.5
C5B—C6B—C1B	123.8 (3)	C13B—C12B—C11B	121.0 (3)
C6A—C5A—H5A	122.1	C13B—C12B—H12B	119.5
C4A—C5A—H5A	122.1	C14B—C13B—C12B	118.8 (3)
C4A—C5A—C6A	115.9 (3)	C14B—C13B—H13B	120.6
N2A—C6A—C1A	104.1 (2)	C12B—C13B—H13B	120.6
C5A—C6A—N2A	132.0 (3)	C11A—C12A—H12A	119.4
C5A—C6A—C1A	123.9 (3)	C13A—C12A—C11A	121.1 (3)
O1B—C10B—C9B	119.9 (3)	C13A—C12A—H12A	119.4
O1B—C10B—C11B	121.4 (3)	C16B—C15B—H15B	120.4
C9B—C10B—C11B	118.7 (3)	C14B—C15B—C16B	119.1 (3)
C6A—C1A—N1A	111.6 (3)	C14B—C15B—H15B	120.4
C2A—C1A—N1A	129.8 (3)	C13A—C14A—Cl1A	118.6 (3)
C2A—C1A—C6A	118.6 (3)	C15A—C14A—Cl1A	120.1 (3)
C16B—C11B—C10B	118.8 (3)	C15A—C14A—C13A	121.3 (3)
C12B—C11B—C10B	122.2 (3)	C12A—C13A—H13A	120.5
C12B—C11B—C16B	119.0 (3)	C14A—C13A—C12A	119.0 (3)
C6B—C5B—H5B	121.9	C14A—C13A—H13A	120.5
C6B—C5B—C4B	116.2 (3)	C16A—C15A—H15A	120.4
C4B—C5B—H5B	121.9	C14A—C15A—C16A	119.1 (3)
C16A—C11A—C10A	119.3 (3)	C14A—C15A—H15A	120.4
			
S1B—C9B—C10B—O1B	−15.4 (5)	C9B—C10B—C11B—C16B	143.2 (3)
S1B—C9B—C10B—C11B	165.7 (2)	C9B—C10B—C11B—C12B	−38.8 (5)
S1A—C9A—C10A—O1A	22.5 (4)	C1B—N1B—C7B—S1B	179.7 (3)
S1A—C9A—C10A—C11A	−157.4 (2)	C1B—N1B—C7B—N2B	−0.9 (3)
Cl1B—C14B—C13B—C12B	−179.4 (3)	C1B—C6B—C5B—C4B	−0.4 (5)
Cl1B—C14B—C15B—C16B	−179.9 (3)	C9A—S1A—C7A—N2A	1.5 (2)
Cl1A—C14A—C13A—C12A	−179.1 (3)	C9A—S1A—C7A—N1A	−179.6 (3)
Cl1A—C14A—C15A—C16A	178.5 (3)	C6B—N2B—C7B—S1B	−179.2 (2)
N2B—C8B—C9B—S1B	−0.5 (3)	C6B—N2B—C7B—N1B	1.2 (4)
N2B—C8B—C9B—C10B	−172.8 (3)	C6B—N2B—C8B—C9B	179.8 (3)
N2B—C6B—C5B—C4B	−180.0 (3)	C6B—C1B—C2B—C3B	0.7 (5)
N2A—C8A—C9A—S1A	0.5 (3)	C6B—C5B—C4B—C3B	0.9 (5)
N2A—C8A—C9A—C10A	172.1 (3)	C5A—C6A—C1A—N1A	178.3 (3)
N2A—C6A—C1A—N1A	0.0 (3)	C5A—C6A—C1A—C2A	−0.1 (5)
N2A—C6A—C1A—C2A	−178.3 (3)	C5A—C4A—C3A—C2A	1.1 (5)
N1B—C1B—C6B—N2B	0.4 (3)	C6A—N2A—C7A—S1A	178.3 (2)
N1B—C1B—C6B—C5B	−179.2 (3)	C6A—N2A—C7A—N1A	−0.9 (4)
N1B—C1B—C2B—C3B	179.2 (3)	C6A—N2A—C8A—C9A	−179.1 (3)
O1B—C10B—C11B—C16B	−35.7 (5)	C6A—C5A—C4A—C3A	−1.2 (5)
O1B—C10B—C11B—C12B	142.3 (4)	C6A—C1A—C2A—C3A	−0.1 (5)
N1A—C1A—C2A—C3A	−178.2 (3)	C10B—C11B—C16B—C15B	179.4 (3)
C7A—S1A—C9A—C8A	−1.1 (3)	C10B—C11B—C12B—C13B	−178.5 (3)
C7A—S1A—C9A—C10A	−173.4 (3)	C1A—N1A—C7A—S1A	−178.0 (3)
C7A—N2A—C8A—C9A	0.7 (4)	C1A—N1A—C7A—N2A	0.9 (3)
C7A—N2A—C6A—C5A	−177.5 (3)	C1A—C2A—C3A—C4A	−0.4 (5)
C7A—N2A—C6A—C1A	0.5 (3)	C11B—C16B—C15B—C14B	−1.3 (5)
C7A—N1A—C1A—C6A	−0.6 (3)	C11B—C12B—C13B—C14B	−0.2 (5)
C7A—N1A—C1A—C2A	177.6 (3)	C11A—C16A—C15A—C14A	1.5 (5)
C7B—S1B—C9B—C8B	0.8 (3)	C11A—C12A—C13A—C14A	−0.5 (5)
C7B—S1B—C9B—C10B	173.7 (3)	C2B—C1B—C6B—N2B	179.2 (3)
C7B—N2B—C8B—C9B	−0.2 (4)	C2B—C1B—C6B—C5B	−0.4 (5)
C7B—N2B—C6B—C1B	−0.9 (3)	C2B—C3B—C4B—C5B	−0.7 (5)
C7B—N2B—C6B—C5B	178.7 (3)	C10A—C11A—C16A—C15A	−177.3 (3)
C7B—N1B—C1B—C6B	0.3 (3)	C10A—C11A—C12A—C13A	176.6 (3)
C7B—N1B—C1B—C2B	−178.4 (3)	C4B—C3B—C2B—C1B	−0.1 (5)
C8B—N2B—C7B—S1B	0.8 (3)	C4A—C5A—C6A—N2A	178.5 (3)
C8B—N2B—C7B—N1B	−178.8 (3)	C4A—C5A—C6A—C1A	0.8 (5)
C8B—N2B—C6B—C1B	179.0 (3)	C16A—C11A—C10A—O1A	26.5 (5)
C8B—N2B—C6B—C5B	−1.3 (6)	C16A—C11A—C10A—C9A	−153.6 (3)
C8B—C9B—C10B—O1B	156.5 (3)	C16A—C11A—C12A—C13A	0.8 (5)
C8B—C9B—C10B—C11B	−22.5 (5)	C16B—C11B—C12B—C13B	−0.5 (5)
C8A—N2A—C7A—S1A	−1.5 (3)	C12B—C11B—C16B—C15B	1.3 (5)
C8A—N2A—C7A—N1A	179.3 (3)	C13B—C14B—C15B—C16B	0.6 (5)
C8A—N2A—C6A—C5A	2.2 (6)	C12A—C11A—C10A—O1A	−149.3 (3)
C8A—N2A—C6A—C1A	−179.8 (3)	C12A—C11A—C10A—C9A	30.6 (5)
C8A—C9A—C10A—O1A	−148.5 (3)	C12A—C11A—C16A—C15A	−1.4 (5)
C8A—C9A—C10A—C11A	31.6 (5)	C15B—C14B—C13B—C12B	0.2 (5)
C9B—S1B—C7B—N2B	−0.9 (2)	C13A—C14A—C15A—C16A	−1.1 (6)
C9B—S1B—C7B—N1B	178.6 (3)	C15A—C14A—C13A—C12A	0.6 (5)

### (Benzo[4,5]imidazo[2,1-b]thiazol-2-yl)(4-chlorophenyl)methanone Hydrogen-bond geometry (Å, º)

*Cg*1, and *Cg*7 are the centroids of of atoms S1*A*/C7*A*/N2*A*/C8*A*/C9*A* and C1*B*–C6*B*, respectively.

**Table:**

*D*—H···*A*	*D*—H	H···*A*	*D*···*A*	*D*—H···*A*
C8*A*—H8*A*···N1*A*^i^	0.93	2.30	3.206 (3)	165
C8*B*—H8*B*···N1*B*^ii^	0.93	2.31	3.212 (3)	163
C13*B*—H13*B*···*Cg*1^ii^	0.93	2.88	3.551 (4)	130
C16*A*—H16*A*···*Cg*7^iii^	0.93	3.00	3.832 (4)	150

Symmetry codes: (i) *x*, −*y*+1/2, *z*−1/2; (ii) *x*, −*y*+3/2, *z*−1/2; (iii) −*x*+1, *y*−1/2, −*z*+3/2.
